# Prevention and Arrest of Lice-Borne Diseases by New Methods of Disinfection

**Published:** 1919-01

**Authors:** 


### *Prevention and Arrest of Lice-Borne Diseases by New Methods of Disinfection.* By Colonel William Hunter. *The Lancet*, Sept. 14 and 21, 1918.

The subject of the lecture delivered by Col. Hunter relates to the prevention of typhus relapsing fever and trench fever through lice.

The principle of primary importance, in connection with disinfection against lice borne disease, is the necessity of carrying out disinfection on the largest possible scale and in the shortest period of time; the object in view being to kill off, within a period of a few days, every living thing in the clothes and on the body that may have contracted infection and be capable of conveying such infection.

This principle is carried out with a rapidity and efficiency unapproached by any other method, namely: by the current steam railway van disinfector. This type of disinfection was devised by Lt. Col. G. Stammers and first put into use in Serbia, March, 1915, to deal with the great epidemics of typhus, relapsing fever, and typhoid fever then raging in that country. It was subsequently introduced in Egypt in February, 1916, with improvements increasing its efficiency. The principle of its action is disinfection by "current steam" supplied and directly discharged into one or two railway vans by the steam engine.

The cost of adaptation is only 30 or 40 pounds, the working simple and easy. From personal knowledge Col. Hunter says that each van, if fully employed, is capable of carrying out disinfection at the rate of 500 kits with 100 blankets, and 500 kits with 1000 blankets and 500 overcoats every two hours, or disinfecting over 18,000 kits, with 36,000 blankets and 18,000 overcoats in nine days.

Divisions of troops can be dealt with at the rate of 16,000 in 12 days. This rapidity of work does not apply to all railway van disinfectors as such, but only to the original Stammer's van as modified in Egypt. The steam power is so great, and the methods of dispersion so arranged, that it is possible to disinfect bundles of clothing in bulk without unpacking.

The double van was found to work with absolute uniformity, the exposure given being an hour for van full up with kits, and 15 minutes for overcoats which were thrown in loosely, and in every case the eggs as well as the lice were killed. This is not mere conjecture; it is founded on verified tests. A self-registering thermometer, even when enclosed in its case and put into the center of the largest bundle, registered temperatures of 102 degrees to 105 degrees C. within half an hour after the steam was turned on from the engine into the van.

A study of the results obtained in Egypt showed the closest relationship to exist between the incidence of the disease and the amount of disinfection carried out in the unit affected. In one place in Egypt only three cases of relapsing fever occurred in the next 12 months instead of over 50 in the month when the disinfection was carried out.

If the disinfection can be carried out rapidly in the whole of the unit the disease is thoroughly arrested. The reason emphasized is that disinfection, to be effective in arresting a louse-borne disease in any area, must be applied to the whole of the individuals in that area within a maximum period of 2 to 3 weeks (i.e. the incubation of lice-borne disease); otherwise the disease continues to occur, men being reinfected with infected lice from those who have not been disinfected.

While stationary disinfection can only be used for those in the neighborhood, the van disinfector can carry out this larger number of disinfections daily over an area of 100 miles or more, whenever and wherever they are required.

*Construction of Van Disinfectors.*

The van disinfectors, which proved most efficient and were found most useful in Serbia, were devised and formed from a wooden and a meat van.

The adaptation of the wooden van consisted in placing a narrow steam pipe round the floor and close to its sides, connected at the end of the van with a similar pipe coming from the boiler of a small engine. The pipe had a few apertures of considerable size and several feet apart to let the steam into the van. Movable bars of wood stretching across the van were devised for the spreading out of the clothes. The meat van was also very easily changed into a disinfector. A perforated ventilating pipe near the roof proved most convenient to convey the steam into the van. Since the van was lined with tin, this aided greatly in obtaining rapid heating of the walls. Meat-hooks were used to hang clothes upon. This very simple adaptation required only 3 days. On trial the temperature was found to rise to 97° C, in about half an hour.

*Special Features of the Van Disinfector.*

This third railway van disinfector consisted of one or two meat vans adapted to the disinfecting process, and an old ordinary engine capable of developing steam and conveying the vans along the railway lines.

The steam pipe used lastly in the vans was a wide one, 2 or 3 inches in diameter, placed around the floor of the van, and about 2 feet from the sides. It had a series of small apertures, 1/8 inch in diameter, placed in two rows along the upper convexity of the pipe, at intervals of 6 inches, and alternately right and left. Wooden racks, with open shelves, 3 feet wide and 3 feet deep, were placed around the van, the lowest shelf being 2 feet from the floor, directly over the steam pipe.

The clothes did not require to be spread out, but simply placed in their original bundles on the racks.

When the steam was turned on the van was filled at once with dense volumes of steam, issuing with great force in a finely divided spray, in all directions.

The walls of the van became burning hot in 10 minutes.

The temperature rose to 102° C. in 10 to 15 minutes, and to 105° C. (221° F.) in about 20 minutes in the center of the largest bundles.

No attempt was made to develop any pressure in the van, steam being allowed to escape below and around the doorways.

As they came out of the van the clothes were not wet with warm water, but moist with steam, and, if exposed at once to the air, they dried in a minute or two.

*Disinfection by Means of "Barrel Disinfectors".*

The needs for regimental disinfection are most simply and sufficiently met with by the "barrel disinfectors". From the time of its first introduction and trial in Serbia, in March 1915, it has proved a most effective protection against typhus and recurrent fever.

*Nature of Barrel Disinfectors.*

A sound wine or water barrel is taken. Through the bottom of this one large central hole is made, with a circle of five or six holes around it, through which the steam can enter the barrel, since it stands on a circular boiler. To prevent any escape of the steam between the boiler and the bottom of the barrel, a narrow sausage ring filled with sand is placed between the boiler and barrel. The weight of the barrel presses this down, forming an efficient valve. A small wooden or wicker-work frame is placed inside the barrel, about 9 inches above the holes, to keep the clothes in the barrel away from these.

The barrel is provided with a heavy lid, the object of which is to retard the escape of steam; the purpose being disinfection by steam.

The temperature reaches 100° C; after the barrel is thoroughly heated, the time required for disinfection of clothing is one hour.

*Use of Barrel Disinfectors in the Field.*

One such disinfector to each company is capable of disinfecting the men of the company every 2 to 3 weeks.

*Typhus and Relapsing Fever in Serbia, 1915.*

When the British Sanitary Mission of 1915 set to work in Serbia, epidemics were spreading out most rapidly. 36 000 were sick in hospitals, of which 15 000 were fever cases.

During March, 1915, the numbers of cases of typhus in hospitals rose from 3 700 to 8 100; mortality was about 40 per cent.

A "Programme of Prevention" was elaborated and resorted to immediately. It included: (1) The establishment of a big quarantine and cleansing station, just behind the three main armies, to break the lines of infection between the troops and the rest of the country.

(2) The stopping of all railway communication for a time and all leave from the Army.

(3) The widespread use of a simple disinfecting process: namely, of the barrel disinfectors, which were to be supplied broadcast to all towns and villages in all parts of the country.

*The English Sanitary Train in Serbia* began to work in the middle of April, 1915. It consisted of coaches specially adapted for inoculation and disinfection, and bath vans formed from meat vans. It was splendidly fitted to meet the urgent needs of the situation, and was certainly responsible, in large measure, for the rapidity with which the epidemics were checked.

*Course of the Epidemics of Typhus and Relapsing Fever in Serbia.*

The relationship between the introduction of the preventive and disinfecting measures and the course of the epidemics is so close that there is no doubt as to their connection as cause and effect.

The official charts show quite distinctly that the arrest of the epidemics was, in the first place, related to the great preventive measure of suspension of railway traffic; but the subsequent subsidence was largely influenced by the adoption of barrel disinfectors. In the space of one month the measures, generally adopted by that time, occasioned a fall of 42 per cent. in the number of typhus cases in the large army hospitals of Kragujewatz.

*Prevention of Trench Fever.*

The connection between trench fever and lice seems most probable, but has never been definitely proved. It seems likely that trench fever can be prevented by strict and regular regimental disinfection.

The author's conclusion, subsequent to his own experiences in the Eastern Areas of the war, is that the group of lice-borne diseases is far more amenable to arrest and prevention by simple measures of disinfection, as van and barrel disinfectors, easily improvized and widely applicable, than by complicated apparatus of autoclave nature.

